# A perspective on the recent progress in solution-processed methods for highly efficient perovskite solar cells

**DOI:** 10.1080/14686996.2016.1226120

**Published:** 2016-10-12

**Authors:** Ashwith Chilvery, Sanjib Das, Padmaja Guggilla, Christina Brantley, Anderson Sunda-Meya

**Affiliations:** ^a^ Department of Physics and Dual Engineering, Xavier University of Louisiana, New Orleans, LA, USA;; ^b^ Department of Electrical Engineering and Computer Science, University of Tennessee, Knoxville, TN, USA;; ^c^ Department of Physics, Alabama A&M University, Normal, AL, USA;; ^d^ AMRDEC, Redstone Arsenal, Huntsville, AL, USA

**Keywords:** Solution-processing, perovskite thin films, perovskite solar cells, 50 Energy Materials, 209 Solar cell / Photovoltaics

## Abstract

Perovskite solar cells (PSCs) were developed in 2009 and have led to a number of significant improvements in clean energy technology. The power conversion efficiency (PCE) of PSCs has increased exponentially and currently stands at 22%. PSCs are transforming photovoltaic (PV) technology, outpacing many established PV technologies through their versatility and roll-to-roll manufacturing compatibility. The viability of low-temperature and solution-processed manufacturing has further improved their viability. This article provides a brief overview of the stoichiometry of perovskite materials, the engineering behind various modes of manufacturing by solution processing methods, and recommendations for future research to achieve large-scale manufacturing of high efficiency PSCs.

## Graphical abstract

**Figure:**
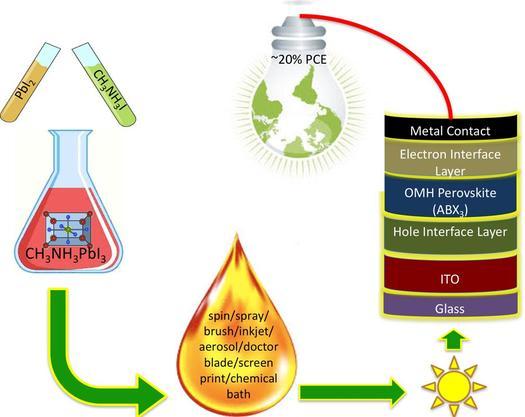

## 1. Introduction

**Figure 1.**
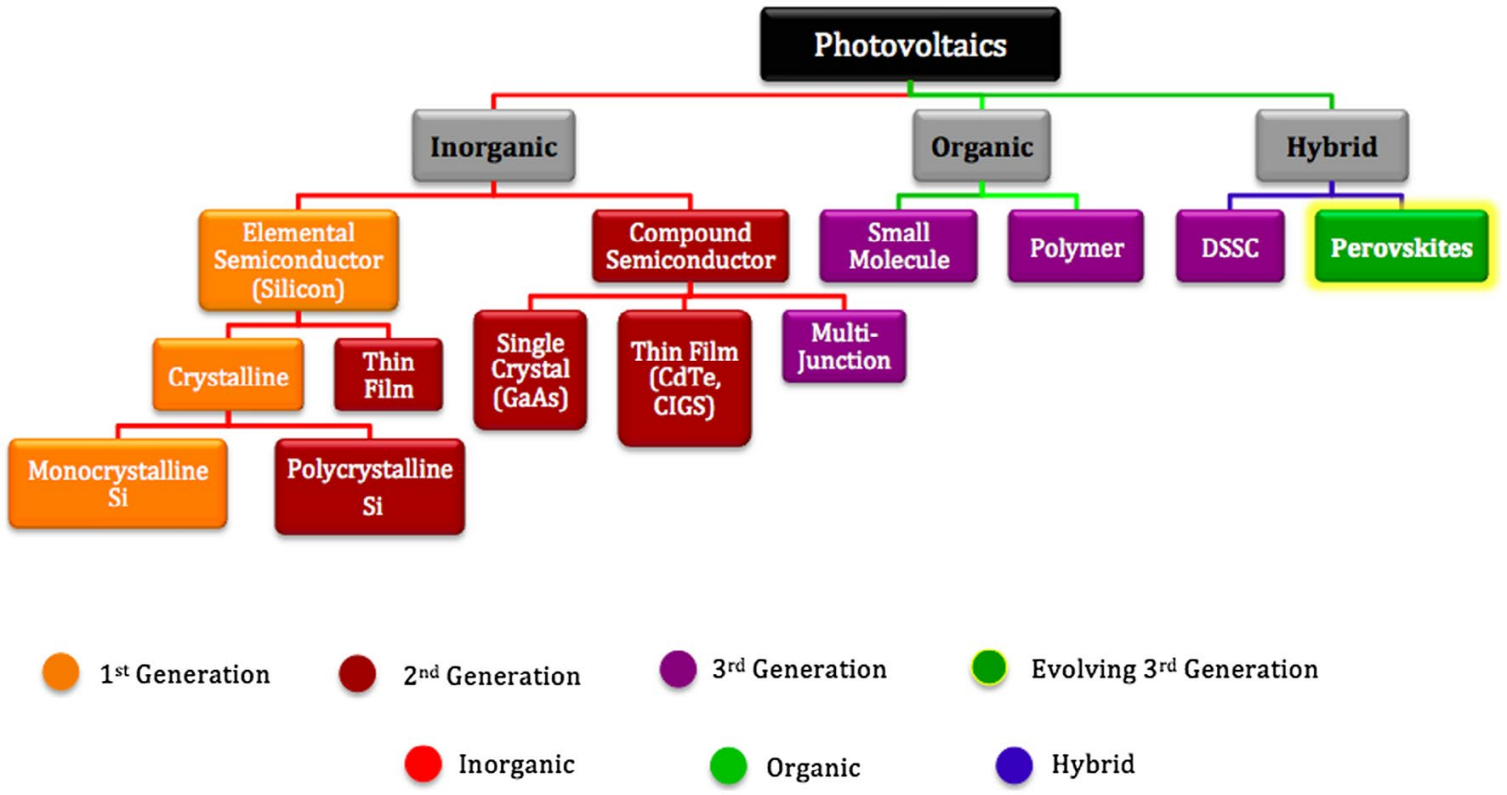
Taxonomy of PV technologies. Adapted from [13].

Photovoltaic (PV) technology plays a significant role in conserving fossil fuels, with the introduction of innovative technologies and functional materials, e.g. organometal halide (OMH) perovskites. Solar cells based on OMHs – perovskite solar cells (PSCs) – have received a lot of attention from the scientific community in recent years due to their flexibility, low weight, and low cost.[1–3] A detailed classification of the major PV technologies is given in Figure 1.

Since the advent of OMH-based PSCs in 2009 through the seminal work of Kojima et al*.* these devices have displayed best-in-class performances and superior efficiencies, progressing from 3.8% to 22.1% in 6–7 years.[4–7] The three-dimensional (3D) frameworks of the organometallic halides incorporated in PSCs have proven to be excellent transformers of abundant solar photons to electrons. OMHs, with a stoichiometry of perovskite crystal, have some excellent properties, such as long electron-hole diffusion lengths (>100 nm) and carrier lifetimes, direct band gap with large absorption coefficients, and low-cost solution-based processing capabilities, that made them the best functional materials for solar cells.[8–12]

In addition to these excellent optoelectronic characteristics, the solution-processing capability of various PSC device layers (hole transport, electron transport and active perovskite layers) makes the methodology very promising for roll-to-roll fabrication. This article details the achievements made possible by PSCs through various solution-processed deposition techniques and the engineering challenges associated with those techniques.

## 2. Organometal halides and their prospects

**Figure 2.**
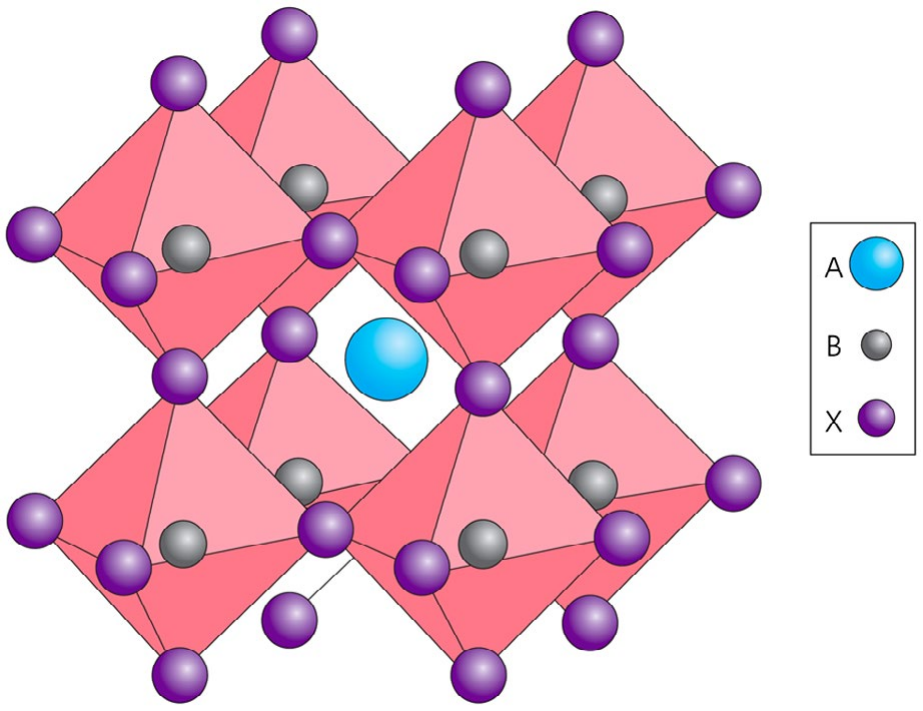
A typical perovskite crystal structure. Reprinted from [15] with permission from Macmillan Publishers Ltd.

OMHs are typically perovskite compounds with an ABX_3_ crystal structure, where A is an organic cation (e.g. methylammonium (MA)), B is a metal cation (Pb or Sn), and X is an anion (Cl, Br, or I) that binds them. Figure 2 illustrates the octahedral symmetry of a cubic perovskite crystal structure. In an ideal cubic perovskite structure, the large A cation is in 12 coordination and slightly smaller B cations occupy the octahedral holes formed by the large X anions. The OMHs display some unique physical and optoelectronic properties due to the hybrid merging of organic and inorganic materials. The advantages of the inorganic components are thermal stability and very high degree of structural order, while the organic materials contribute to functional versatility, mechanical flexibility, and cost-effective processing. When combined, they overcome many of the problems associated with creating efficient charge conduction in PV cells.[14]

In the last three years there have been significant breakthroughs due to formamidinium (HC(NH_2_)_2_^+^) and tin (Sn^2+^) ions that have enabled progression beyond the conventional methylammonium (CH_3_NH_3_^+^) and lead (Pb^2+^) ions. A power conversion efficiency (PCE) of more than 20% was achieved after fine-tuning the band gap of perovskite by placing a formamidinium ion in the organolead trihalide perovskite structure.[5] As a step towards replacing hazardous lead in PSCs, several researchers were able to fabricate tin-based PSCs with encouraging PCEs in the range of ~6%.[16,17] Since these compounds have their band gaps spanning most of the visible region, the ability to tune their electronic structures could further optimize their performance in PV applications. Further, the concept of tolerance factor is considered as a guide to the cubic structures of perovskites. A recent study on the tolerance factors revealed that there are over 600 undiscovered amine–metal–anion permutations based on halides and molecular (organic) anions suitable for PV applications.[18] Typically, the dielectric permittivity of the perovskite compounds in PSCs is higher than that of the organic semiconductors in organic solar cells due to their hybrid mixture of polarized ionic compound, inorganic anion, and organic cation, which collectively contribute to a swift and long-range charge transport via band structure or polaron hopping.[19] Due to this phenomenon, the excitons experience lower binding energies and higher Bohr’s excitonic radius, thereby achieving superior charge transports.

## 3. Perovskite solar cells: device architectures

**Figure 3.**
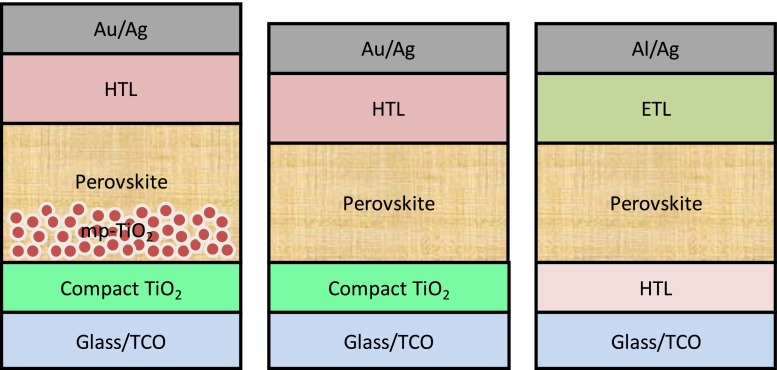
Typical architectures of PSCs – mesoporous (left), conventional planar (middle) and inverted planar (left); HTL – hole transport layer, ETL – electron transport layer, TCO – transparent conductive oxide (e.g. indium tin oxide).

Perovskite materials exhibit excellent optoelectronic properties and superior device performance via two key device architectures – mesoscopic and planar – as illustrated in Figure 3. OMH perovskite absorbers were first demonstrated by Kojima et al. [4] in a dye-sensitized solar cell architecture; an efficiency of 3.8% was achieved by replacing the dye with perovskite^[4]^. In 2011, Im et al*.* [20] optimized and incorporated CH_3_NH_3_PbI_3_ quantum dots on a nanocrystalline TiO_2_ surface, which yielded an efficiency of 6.54% on electrochemical reactions with iodine based redox electrolyte^[20]^. In 2012, Kim et al. [21] deposited nanoparticles of CH_3_NH_3_PbI_3_ on the submicrometer-thick mesoscopic TiO_2_ film exhibiting a panchromatic absorption of visible light with improved stability, leading to an excellent photocurrent density of 17.6 mA cm^–2^ and a PCE of 9.7%^[21]^. Also in 2012, Lee et al. [22] replaced the traditional *n*-type, mesoporous TiO_2_ with insulating aluminum oxide (Al_2_O_3_) and achieved a PCE of 10.9% in a single-junction device under standard illumination (AM 1.5, 100 mW cm^–2^)^[22]^. Etgar et al. [23] investigated a hole transport layer (HTL)-free mesoscopic CH_3_NH_3_PbI_3_ heterojunction solar cell, achieving an encouraging PCE of 5.2%^[23]^. Further, a 3D nanocomposite of mesoporous TiO_2_ with CH_3_NH_3_PbI_3_ as a light harvester and hole conductor substantially improved the PV parameters by achieving a PCE of 12% with a high open circuit voltage (*V*_OC_) of 0.997 V, short-circuit current density (*J*_SC_) of 16.5 mA cm^–2^, and a fill factor (FF) of 0.727.[24]

MA iodide (CH_3_NH_3_I) based perovskites, MAPbI_3_ and MAPbI_*x*_Cl_3-*x*_, are the two perovskite materials that have been extensively used as the absorber (also call intrinsic) layer in PSCs as they possess properties that are prerequisites to having excellent photovoltaic performance. During the last two years or so, many research groups came up with a series of new perovskite materials with suitable band gap by replacing the organic MA cation by FA cation and/or by tuning the halides, for example, FAPbI_3_.[5,25] One of the big advantages of band gap engineering in perovskites is the production of tandem or multi-junction solar cells as was demonstrated by McMeekin et al. [26].

During the initial evolution of PSCs, the mesoporous layer was thought to be necessary for efficient charge transport and better device performance.[22,27,28] Interestingly, many research groups later showed that the mesoscopic layer is not necessary to achieve higher PCEs; a simple planar architecture is enough to achieve excellent perovskite film coverage and device performance.[29–31] However, it is still under debate as some research groups have shown that incorporating a mesoporous layer can enhance the device stability and reduce the so-called *J*-*V* hysteresis in PSCs.[32–35] Planar PSCs, on the other hand, are simple in configuration and easy to fabricate. They consist of either a conventional (*n*-*i*-*p*) or an inverted (*p*-*i*-*n*) device configuration, as shown in Figure 3,[36–40] both of which are similar to organic photovoltaic (OPV) device architectures. In both conventional and inverted PSCs, the perovskite layer is sandwiched between hole and electron transport layers. Some of the *p*-type HTLs most often used in conventional PSC architecture are 2, 2′, 7, 7′-tetrakis(*N*, *N*-di-p-methoxyphenyl-amine)-9, 9′-spirobifluorene (spiro-OMeTAD) and poly(triarylamine) (PTAA), while the inverted architecture mostly incorporates poly(3, 4-ethylenedioxythiophene):poly(styrenesulfonate acid) (PEDOT:PSS) as HTL.[25,31,36] Due to its hygroscopic nature, the device stability of inverted PSCs incorporating PEDOT:PSS is concerned. On the other hand, most of the reported conventional and inverted PSCs either used TiO_2_ or fullerene as the electron transport layer. One of the notable alternatives to TiO_2_ is ZnO,[41,42] while PEDOT:PSS can be replaced by NiO_*x*_.[43,44]

The planar solid perovskite films are superior in charge carrier mobilities and photo-generated carrier lifetimes when compared to the mesoporous films.[45] Hence, high-efficiency cells with a single solution-processed solid absorber layer would be advantageous. Though it was reported by Eperon et al. [29] that lower performance in planar devices may arise from pin-hole formation, incomplete coverage of the perovskite resulting in low-resistance shunting paths and lost light absorption in the solar cells,^[29]^ significant advancement has been made in terms of fabrication methods to overcome this problem, thereby achieving excellent device performance.[30,46–48]

## 4. Solution-processed fabrication methods

**Figure 4.**
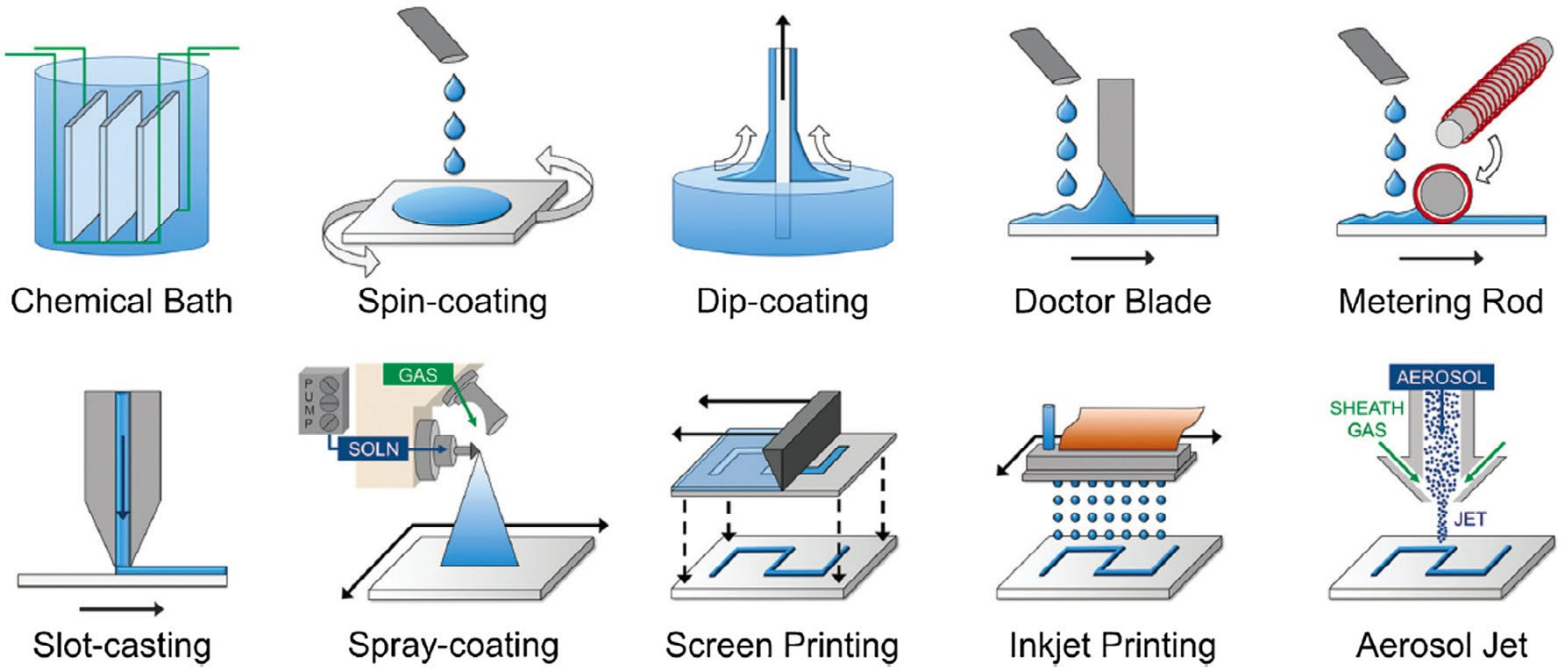
Illustration of various solution processing methods. Reproduced from [66] with permission from The Royal Society of Chemistry.

Irrespective of device architectures, the performance of PSCs is heavily impacted by the perovskite layer itself. In order to achieve high PCE and reproducibility, it is critically important to have a uniform, dense, and pinhole-free perovskite film as noted above. The thinner cells tend to absorb light poorly, whereas the charge carriers cannot travel through to reach the contacts if the absorber layer is too thick due to higher charge recombination, rendering it vital to understand the charge transport kinetics.[49–51] During early developmental stages of PSC technology, it was reported that the vapor deposition achieves better efficiencies than solution-processed devices.[31] Liu et al. [31] reported a dual-source thermal evaporation technique to deposit perovskite thin films, where inorganic PbCl_2_ and organic MA iodide precursors were co-evaporated, and the right stoichiometry was achieved by optimizing the respective precursor evaporation rates^[31]^. This technique resulted in perovskite films with better coverage and uniformity compared to solution-processed films based on the same precursor materials and stoichiometry. However, it is difficult to achieve the right stoichiometry via vapor deposition and optimizing the evaporation rates. Chen et al*.* [52] reported a sequential vapor deposition technique^[52]^, where inorganic and organic precursors were deposited sequentially, and a range of substrate temperatures were used to achieve uniform and dense perovskite films that resulted in a PCE of 15.4%. Though vapor based techniques were found to result in excellent film quality and high PCEs, they are very expensive due to the high vacuum required during the deposition. To reduce the production costs, printing of functional layers by solution processing has evolved into a promising manufacturing technology for flexible electronics and PSCs.[1,53,54] The aforementioned overview on perovskite materials is crucial in understanding the solvent dynamics of various solution-processing PSC fabrication methods such as spin coating, spray-coating, inkjet printing, brush coating, doctor blading, dip-coating, and screen-printing. An illustration of the various solution-processing methods is shown in Figure 4.

In the early days of PSCs, researchers employed one-step spin-coating technique to deposit perovskite films,[29,38,55] where a solution mixture of organic and inorganic precursors is spin-coated, followed by annealing at a certain temperature to drive the reaction. Since these hybrid materials have fast crystallization times, this process usually yields a high density of pinholes in the coated perovskite film, reducing the amount of photogenerated charges, and hence leads to lower short-circuit current density (*J*_SC_), and lower PCE. In order to improve the film coverage, a two-step coating technique was developed, where inorganic and organic precursors were coated sequentially, and a temperature of 70–100 °C was used to drive the inter-diffusion to form perovskite films.[27,46] The two-step technique was shown to result in high quality, uniform, and dense perovskite films, and comparable PCEs to those achieved by vapor deposition.[46,56] The performance of solution-processed PSCs was further improved by incorporating a post-washing step, where an orthogonal solvent that does not dissolve perovskite but is miscible with the host solvent was subsequently coated. Consequently, both the film coverage and uniformity were further improved, and a PCE of beyond 16% was achieved.[57]

One of the biggest advantages of sequential deposition of inorganic and organic precursors is that it prevents fast crystallization of OMH perovskites, due to the fact that the precursor materials need time to interdiffuse, which in turn provides ample nucleation time, yielding better-coverage films with larger perovskite crystals. To further slow down the crystallization, Yang et al. [46] incorporated an intermediate air-exposure step between film deposition and annealing^[46]^. Based on this procedure, the researchers were able to achieve an average PCE of more than 16.0%. Several research groups reported air-exposure, as an intermediate step or during annealing, to be beneficial for both device performance and stability.[30,37,58–60]

Some of the other notable modified two-step coating methods include: (a) incorporation of more than one organic or inorganic precursors;[36,61] (b) use of additive in inorganic precursor solution;[62] and (c) use of molecular self-assembly by using a binary solvent system for inorganic precursor.[63] Despite resulting in uniform film coverage and excellent PCEs, the two-step coating techniques discussed above incorporate spin-coating, and hence are not compatible with large-area or large-scale processing used in industrial-scale roll-to-roll (R2R) manufacturing of PSCs.

To address the manufacturing challenges of high-performance PSCs, several scalable techniques such as slot-die coating, doctor blading, ink-jet printing, and ultrasonic spray coating have been developed or employed. Deng et al. [64] reported on the fabrication of efficient PSCs by a simple, high-throughput, and economical doctor-blading method, compatible with R2R fabrication for large-scale production^[64]^. The fabricated PSCs had an architecture of ITO/PEDOT:PSS/MAPbI_3_/[6,6]-phenyl-C61-butyric acid methyl ester (PC_60_BM)/C_60_/2,9-dimethyl-4, 7-diphenyl-1,10-phenanthroline (BCP)/Al. In the coating process, the precursor solution was dropped onto the PEDOT:PSS-coated indium tin oxide (ITO) glass substrates, and swiped linearly by a glass blade at a speed of 0.75 cm s^–1^. The substrates were held at a temperature of 125 °C during coating, and the thickness of the resultant perovskite films was controlled by varying the perovskite precursor solution concentration and depth of the blading channel. By controlling the stoichiometry and thickness of the active perovskite layer, a peak PCE of 15.1% was achieved. Razza et al. [65] described the development and optimization of air-assisted blade coating of PbI_2_ layers for the fabrication of PSCs exhibiting an excellent PCE of 10.4% from a 10.1 cm^2^ active area^[65]^. Two major advantages of the blade deposition technique are that it reduces the materials used, as a wire-wound metering rod can replace the blade, where the diameter of the wire on the rod controls the amount of coating.

Another promising technique for economical printing of functional materials is inkjet printing, widely considered to be material-conserving as well as one of the fastest deposition techniques on large area substrates for optoelectronic and photonic devices.[67,68] In 2014, Wei et al. [69] demonstrated planar PSCs by an inkjet printing technique with a precisely controlled pattern and interface^[69]^, where ink constituted from carbon and MAI transformed PbI_2_ to MAPbI_3_ *in situ*, creating an interpenetrating interface between MAPbI_3_ and a C electrode with minimal charge recombination. The carbon-based planar (fluorine doped tin oxide (FTO)/TiO_2_/MAPbI_3_/C) PSCs printed via the inkjet method have achieved PCEs as high as 11.6%. Similarly, Li et al. [70] have successfully deposited a flat and uniform CH_3_NH_3_PbI_3_ layer on mesoscopic TiO_2_ film with optimized table temperature and ink composition, and this device exhibited a high PCE of 12.3%^[70]^. Mei et al. [71] reported a fully printable PSC with a PCE of 12.8%, where they used a double layer of mesoporous TiO_2_ and ZrO_2_ as a scaffold infiltrated with perovskite, without requiring a hole-conducting layer^[71]^. The devices were also found to be stable for more than 1000 h in ambient atmospheres under full sunlight. Compared to other scalable techniques, inkjet printing is valuable in that it can even print the top metal electrode and does not require any high vacuum-processing step.[72] Zhang et al. [73] fabricated a PSC by screen-printing and achieved a PCE of 11%^[73]^. It used a mesoporous graphite/carbon black counter electrodes using flaky graphite of different sizes in hole-conductor-free mesoscopic PSC by the screen-printing technique. Hwang et al. [74] reported fully slot-die coated PSCs (ITO/ZnO/MAPbI_3_/P3HT/Ag) using a 3D printer^[74]^. To prevent the formation of overgrown crystals as well as pinholes, they employed an external N_2_ gas-quenching effect to control the drying of PbI_2_ films, thereby resulting in a peak PCE of 11.96%.

Some research groups have also investigated on ultrasonic spray-coating (USC) technique, which is an established and scalable variant of traditional spray techniques. Some of the advantages of USC technique are its formation of picoliter-sized droplets, directional control of the coating using an inert gas providing large-area uniform coverage for thin films, as well as a potential for depositing continuous layers.[75] The ultrasonic nozzle, usually vibrating at a high frequency (e.g. 120 kHz), prevents clogging of the solution at the nozzle-head and produces micrometer-size droplets which minimize the dissolution of underneath layers. In 2014, Barrows et al. [76] reported an efficiency of up to 11% for mixed halide (MAPbI_*x*_Cl_3-*x*_) inverted PSCs (ITO∕PEDOT:PSS∕MAPbI_3-*x*_Cl_*x*_/PCBM/Ca/Al), fabricated by an USC method^[76]^. The study also explored the role of the temperature of the substrate during spray casting, the volatility of the casting solvent, and the post-deposition annealing in the efficiency of the resultant solar cells. Li et al*.* [77] also reported on a facile spray fabrication of a homogeneous and flat MAPbI_3_ for mesostructured PSCs^[77]^. This study also compared the spray-assisted method with the dipping process and observed that it provides pinhole-free and smoother surface, remarkably reducing the RMS roughness from 82 to 16 nm.

**Figure 5.**
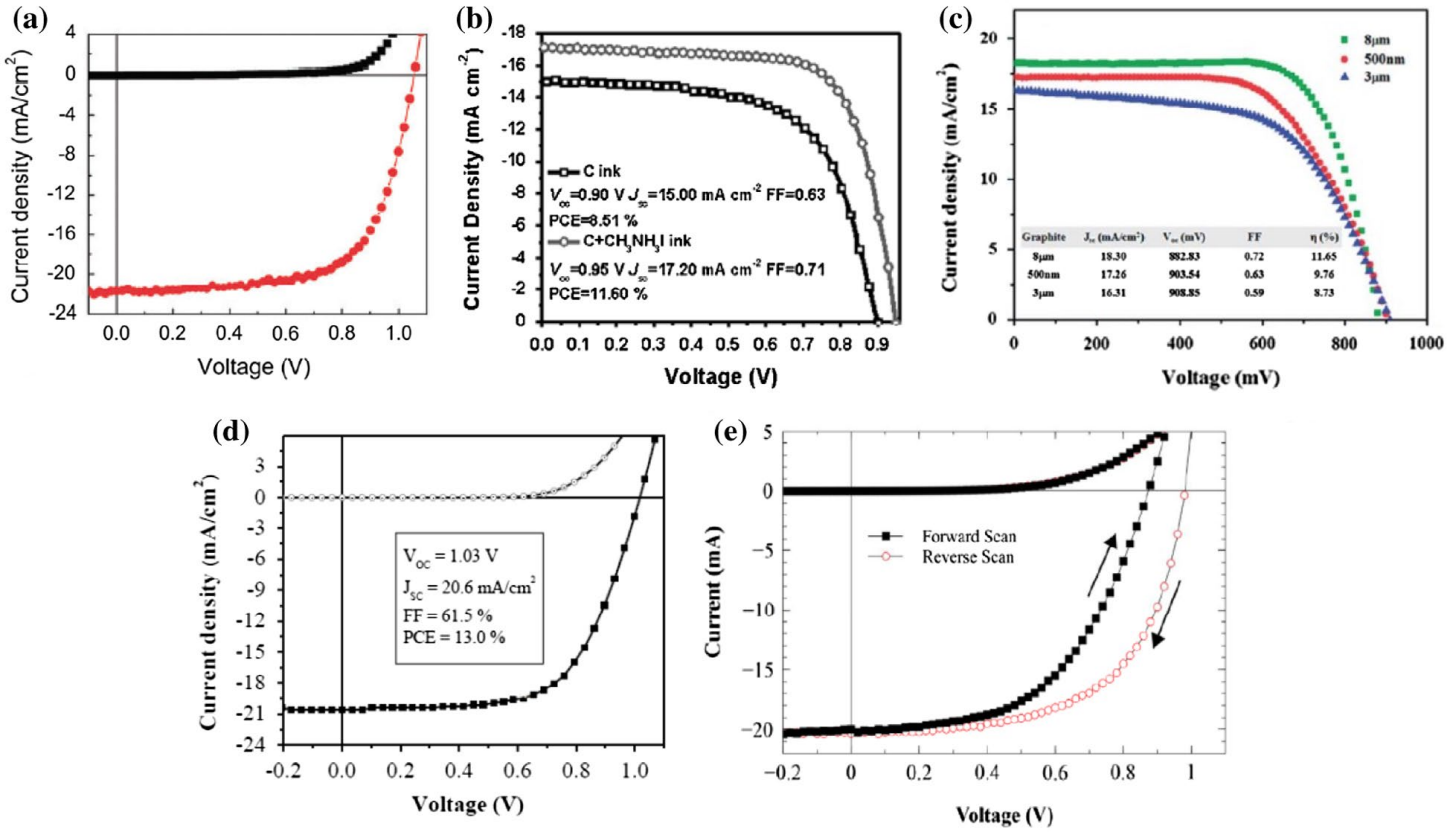
Current density-voltage (*J*-*V*) curves of various solution-processed devices: (a) blade-coated planar PSC (Reproduced from [64] with permission from the Royal Society of Chemistry); (b) inkjet-printed FTO/TiO_2_/MAPbI_3_/C planar PSC (Reproduced from [69] with permission from John Wiley and Sons, Inc.); (c) screen-printed PSC (Reproduced from [73] with permission from the Royal Society of Chemistry); (d) ultrasonic spray-coated planar PSC (Reproduced from [78] with permission from ACS publishers); and (e) slot-die coated PSC (Reproduced from [74] with permission from John Wiley and Sons, Inc.).

In 2015, Das et al*.* [78] further optimized the USC process, where a solution mixture of MAI and PbCl_2_ was spray-coated to form highly uniform, dense, and high-quality perovskite (MAPbI_3-*x*_Cl_*x*_) thin films^[78]^. PSCs with conventional architecture (ITO/TiO_2_/MAPbI_3-*x*_Cl_*x*_/Spiro-OMeTAD/Ag), fabricated by optimizing process conditions, exhibited PCEs as high as 13.0% on glass substrates. Most importantly, flexible PSCs on polyethylene terephthalate (PET) substrates showed a reasonable peak PCE of 8.1%. As a comparison of performances of PSCs based on different scalable, solution-processed deposition techniques, Figure 5 depicts various representative current density vs. voltage (*J*-*V*) characteristics, and Table 1 details the overall device performances.

**Table 1.** Comparison of device performances of PSCs fabricated by various scalable techniques.

Device architecture	Coating technique	*J*_SC_[mA/cm^2^]	*V*_OC_[V]	FF[%]	PCE[%]	Reference
FTO/bl-TiO_2_/mp-TiO_2_/perovskite/PTAA/Au	Two-step spin-coating	24.70	1.06	77.5	20.20	[5]
ITO/ZnO/MAPbI_3_/P3HT/Ag	Slot die-coating	20.38	0.98	59.9	11.96	[74]
ITO/PEDOT:PSS/MAPbI_3_/PCBM/C_60_/BCP/Al	Doctor blading	21.80	1.05	69.2	15.10	[64]
FTO/TiO_2_/CH_3_NH_3_PbI_3_/Spiro-OMeTAD/Au	Blade coating	18.90	1.00	70.5	13.30	[65]
ITO/TiO_2_/MAPbI_3-*x*_Cl_*x*_/Spiro-OMeTAD/Ag	Spray-coating	20.60	1.03	61.6	13.00	[78]
FTO/TiO_2_/MAPbI_3_/Spiro-OMeTAD/LiTFSI/Au	Inkjet printing	19.55	0.910	69	12.3	[70]
FTO/TiO_2_/MAPbI_3_/C	Inkjet printing	17.20	0.95	71.0	11.60	[69]

In addition to the solution-processed techniques discussed so far, other notable techniques include chemical bath deposition (CBD) and aerosol jet. CBD is a direct growth technique, where the growth strongly depends on the duration of deposition, controlled composition and reaction of reagents in solution. The CBD technique has been used to deposit high-quality TiO_2_ layers at room temperature for high-performance PSCs.[79] One of the major advantages of CBD is that it yields highly uniform and reproducible films. Aerosol jet, like ink-jet printing, has the ability to deposit multilayer films without requiring lithographic techniques, and is a no-waste technique. It has been used to deposit active layers in polymer solar cells and can be used for PSCs as well.[80]

From the beginning of organic PV production until today’s PSCs, spin-coating has been the favorite method to realize superior efficiencies. Its contribution to thin films is incomparable because of its ability to produce ultrathin films with great uniformity and achieve superior PCEs.[3,23,41,81–84] However, spin-coating is only suitable for small area deposition on a flat substrate, which would limit the commercialization of PSCs. Hence, PSCs provide a wider scope for researchers to either develop or demonstrate a variety of new methods that are cost-effective, large-area, and R2R compatible, and more importantly, have excellent efficiency.

As PSC technology becomes more established, further improvement in device performance can be expected. However, successful and timely commercialization of this technology to replace already-existing but expensive PV technologies depends on how some of the critical issues, such as hysteresis, lead toxicity, and stability, are addressed.

## 5. Conclusions

Less than a decade of engagement with hybrid organic/inorganic PSCs has led to new frontiers of science and technology. The scientific research community predict that this emerging technology will continue to see further increase in efficiency in coming years. However, some of the urgent issues associated with PSCs, such as lead toxicity, poor stability, and device performance hysteresis must be addressed before commercialization is possible. Studies have responded to toxicity concerns by focusing on tin (Sn) and germanium (Ge) based devices to avoid the use of poisonous lead (Pb), while optimizing material properties for greater efficiencies. More research efforts are needed to understand the origin of hysteresis and how to reduce it. Over 600 different combinations of materials recommended for investigation have the potential to reveal new knowledge that could alter the dynamics of the devices and address all concerns.

The solution-processing methods discussed above are currently the most successful methods for achieving a cheaper, more durable, and scalable PSC technology. These methods are promising and research efforts are focused on searching for a perfect fabrication methodology that optimizes the performance and efficiency of any PSC device.

## Disclosure statement

No potential conflict of interest was reported by the authors.

## Funding

This work was supported by U.S. Department of Homeland Security – BS award [2010-ST-062-000034].
